# Long-term LDL cholesterol burden and stroke: mechanistic insights and emerging strategies for personalized lipid management

**DOI:** 10.3389/fneur.2026.1836134

**Published:** 2026-04-28

**Authors:** Zhuanfang Li, Lan Ye, Jie Li, Yinping Wang, Jun Yang

**Affiliations:** 1 Department of Neurology, The First Affiliated Hospital of Chongqing Medical University, Chongqing, China; 2 Department of Operation Center, Huashan Hospital, Fudan University, Shanghai, China

**Keywords:** atherosclerosis, cumulative exposure, ischemic stroke, low-density lipoprotein cholesterol, PCSK9 inhibitors, precision medicine

## Abstract

Low-density lipoprotein cholesterol (LDL-C) is a causal and modifiable driver of atherosclerotic cardiovascular disease and an important contributor to atherosclerotic ischemic stroke. Increasing evidence suggests that cumulative LDL-C exposure—the combined effect of LDL-C magnitude and duration over time—captures vascular injury more accurately than a single measurement obtained at one clinical encounter. This review uses cumulative LDL-C exposure as an organizing framework for stroke prevention. We summarize current approaches to quantifying long-term LDL-C burden, review epidemiological evidence linking cumulative exposure to incident and recurrent ischemic stroke, and discuss biological mechanisms that may explain these associations. We then examine how lipid-lowering therapies, including statins, PCSK9 inhibitors, and small-interfering RNA-based agents, may reduce cumulative vascular injury. Particular attention is paid to the clinical challenge of balancing ischemic benefit against potential hemorrhagic vulnerability in selected high-risk phenotypes, such as patients with probable cerebral amyloid angiopathy or multiple lobar cerebral microbleeds. We propose a precision-management framework that integrates longitudinal lipid exposure, stroke subtype, genetics, neuroimaging, and dynamic risk assessment. By shifting attention from static lipid values to lifelong exposure, cumulative LDL-C burden may offer a more coherent basis for individualized cerebrovascular prevention.

## 1 Introduction

Stroke remains a major global cause of death and long-term disability, and ischemic stroke accounts for the large majority of cases (1, 2). Recent Global Burden of Disease 2021-based analyses further show that the burden specifically attributable to high LDL-C has continued to increase worldwide. From 1990 to 2021, disability-adjusted life years (DALYs) due to ischemic stroke attributable to high LDL-C increased by 44.55%, underscoring that LDL-related cerebrovascular risk remains a substantial and growing global public health problem (2). Among modifiable vascular risk factors, elevated low-density lipoprotein cholesterol (LDL-C) is central to the pathogenesis of atherosclerotic cardiovascular disease and is especially relevant to large-artery atherosclerotic stroke (3, 4). However, conventional lipid assessment still relies heavily on single LDL-C measurements, which provide only a snapshot of a biological process that unfolds over decades.

This limitation is increasingly important. Vascular injury related to LDL-C is cumulative: both the intensity of exposure and the duration of exposure matter. Recent work has therefore emphasized the concept of cumulative LDL-C burden, an integrated measure of LDL-C exposure across the life course, as a potentially more informative predictor of atherosclerotic disease progression than a single contemporary LDL-C value (5, 6). In cerebrovascular disease, this framework may help explain why patients with apparently acceptable LDL-C levels at one time point can still carry substantial residual risk if their lifetime exposure has been high.

This perspective has direct clinical implications. Even moderately elevated LDL-C may become harmful when sustained over many years, whereas early and persistent LDL-C lowering may shift the entire trajectory of vascular injury. At the same time, increasingly intensive lipid lowering has renewed concern about possible hemorrhagic complications in selected patients, especially those with prior lobar intracerebral hemorrhage, probable cerebral amyloid angiopathy, or extensive cerebral microbleed burden. Against this background, the present review examines how cumulative LDL-C exposure can be measured, how it relates to ischemic and hemorrhagic stroke phenotypes, which mechanisms may underlie these associations, and how this framework may inform safer and more individualized lipid management.

## 2 Quantifying cumulative LDL-C exposure: methods and challenges

A meaningful discussion of cumulative LDL-C burden begins with measurement. LDL-C changes over time in response to aging, diet, treatment intensity, adherence, and genetic background. Accordingly, different analytic approaches have been developed to estimate long- term exposure.

### 2.1 Classical estimation models

The simplest historical approach treats cumulative exposure as a rough product of age and LDL-C level. Although intuitive, this approximation ignores time-varying treatment effects and does not reflect the true shape of LDL-C trajectories. More informative approaches use repeated measurements to estimate a time-weighted average (TWA), cumulative burden above a predefined threshold, or area-under-the-curve-like metrics derived from serial LDL-C values. These methods better capture the longitudinal nature of exposure and have been used in major cohort analyses evaluating cardiovascular and cerebrovascular outcomes (5, 6).

### 2.2 Emerging techniques and remaining limitations

More recent approaches extend beyond repeated averages and attempt to reconstruct life-course exposure more explicitly. These include trajectory-based models, threshold-based burden models, and genetics-informed estimates that infer lifelong LDL-C exposure from inherited variation. Such approaches are conceptually appealing because they align more closely with the biological hypothesis that arterial injury accumulates gradually over time. However, they also have important limitations. Most require dense longitudinal data, assumptions about missing values and treatment adherence, and careful handling of regression dilution bias and survivor bias. In addition, no universal threshold defines “high cumulative LDL-C burden,” and different studies operationalize this concept differently.

For stroke research, this lack of standardization matters. The apparent strength of the association between cumulative LDL-C burden and stroke risk will depend partly on how well long-term exposure is measured. Future studies should therefore move toward integrated models that combine serial LDL-C values with treatment history, adherence, polygenic susceptibility, and imaging markers of vascular injury. At present, a repeated-measure time-weighted average or area-under-the-curve-like metric may be the most practical choice for stroke-oriented research because it can be derived from routinely collected serial LDL-C values while still capturing both intensity and duration of exposure (5, 6). Until such methods are standardized, cumulative exposure should be viewed as a promising but still methodologically evolving construct.

## 3 Epidemiological evidence linking cumulative LDL-C exposure and ischemic stroke

### 3.1 Large-artery atherosclerosis and first stroke risk

The biological and epidemiological rationale for cumulative LDL-C burden is strongest in atherosclerotic disease. Genetic, mechanistic, and clinical evidence consistently supports LDL as a causal factor in atherosclerosis (3, 7). In the cerebrovascular field, prolonged LDL-C exposure is increasingly linked to extracranial and intracranial atherosclerosis, plaque progression, and incident ischemic stroke. Cohort data suggest that cumulative LDL-C burden is associated with asymptomatic intracranial atherosclerotic stenosis and future ischemic stroke even after adjustment for conventional vascular risk factors (8, 9).

A key implication is that single LDL-C measurements may underestimate true vascular risk. Two individuals with the same LDL-C value at one clinic visit may have very different life- course exposure histories, and therefore very different cumulative arterial injury. This distinction is especially relevant to large-artery atherosclerotic stroke, where plaque burden, remodeling, and instability reflect prolonged exposure rather than a single recent laboratory value.

### 3.2 Recurrence risk and secondary prevention

The relevance of cumulative LDL-C exposure persists after stroke has occurred. In secondary prevention, longitudinal LDL-C patterns may be more informative than one-off measurements: lower on-treatment LDL-C has been associated with reduced recurrence risk, and in Chinese patients, lower follow-up LDL-C was linked to fewer recurrent vascular events (10, 11). Together, these findings suggest that post-stroke risk is shaped by lipid exposure over time, particularly in atherosclerotic or other plaque-driven phenotypes, whereas interpretation should be more cautious in cardioembolic and small-vessel-predominant stroke, where the benefit of intensive LDL-C lowering is less certain (8, 9, 12). They also suggest potential population heterogeneity, such that the cumulative LDL-C burden framework may require cautious interpretation in East Asian populations rather than direct extrapolation from Western data (11–13).

## 4 Potential pathophysiological mechanisms

The association between cumulative LDL-C burden and stroke risk is biologically plausible because prolonged lipid exposure affects multiple components of the vascular and neurovascular system.

First, sustained LDL-C elevation promotes plaque initiation, growth, and destabilization. Endothelial dysfunction, oxidative stress, monocyte recruitment, macrophage activation, and foam-cell formation together enlarge the lipid-rich necrotic core and thin the fibrous cap, increasing the likelihood of rupture or erosion (14, 15). In cerebrovascular beds, these processes favor thromboembolism and artery-to-artery embolic events.

Second, oxidized LDL may impair blood–brain barrier (BBB) integrity. Experimental work shows that oxidized LDL can disrupt tight junction proteins, increase endothelial permeability, and amplify oxidative stress, all of which may weaken the neurovascular unit (16, 17). In aging brains, such injury may interact with amyloid-beta and tau-related vascular vulnerability, further aggravating neurovascular dysfunction (18).

Third, prolonged LDL-related oxidative signaling may promote a prothrombotic state. Oxidized LDL can activate platelet scavenger receptors such as CD36 and LOX-1, thereby enhancing platelet activation, aggregation, and thrombo-inflammatory signaling (19, 20). This mechanism may be particularly relevant to recurrent ischemic stroke, where persistent vascular inflammation and thrombogenicity can reinforce one another over time.

Taken together, these pathways suggest that cumulative LDL-C burden is not merely a statistical marker. Rather, it reflects a biologically meaningful process involving chronic endothelial injury, plaque vulnerability, BBB dysfunction, and thrombo-inflammatory amplification.

## 5 Clinical implications and therapeutic strategies

### 5.1 Lipid-lowering therapy and the reduction of future LDL-C burden

If long-term LDL-C exposure drives vascular injury, therapy should aim not only to lower LDL-C now but also to reduce future cumulative burden. This framing helps explain why early, sustained, and sufficiently intensive lipid lowering can yield durable cerebrovascular benefit.

#### 5.1.1 Statins: foundational therapy in secondary prevention

Statins remain the foundation of lipid-lowering therapy because they reduce LDL-C through HMG-CoA reductase inhibition and also improve endothelial function, reduce inflammation, and stabilize plaques. In secondary stroke prevention, the SPARCL trial showed that high-dose atorvastatin reduced recurrent stroke despite a small increase in hemorrhagic stroke, and the Treat Stroke to Target (TST) trial showed that targeting LDL-C below 70 mg/dL after ischemic stroke or transient ischemic attack with atherosclerosis reduced subsequent cardiovascular events compared with a higher target range (21).

Viewed through the cumulative-exposure lens, the value of statins lies not simply in producing a lower LDL-C number at one visit, but in changing the patient’s exposure trajectory over years. This is particularly relevant for patients with large-artery atherosclerosis or other evidence of substantial historical lipid burden, in whom sustained lowering is likely to generate greater absolute benefit.

#### 5.1.2 PCSK9 inhibitors and long-acting RNA-based agents

PCSK9 inhibition offers an especially strong rationale in patients with persistent residual risk despite statins. In the FOURIER trial, evolocumab added to statin therapy lowered LDL-C to very low levels and reduced cardiovascular events, including ischemic stroke, without a clear signal for major safety excess during trial follow-up (22, 23). Extended follow-up in FOURIER-OLE further suggested that achieved LDL-C levels below 20 mg/dL were associated with lower long-term cardiovascular risk without an obvious increase in major safety outcomes, although these analyses were not designed specifically around stroke subtypes (24). Importantly, evidence is no longer limited to individual trials. A 2024 systematic review and meta-analysis showed that PCSK9 inhibitors reduced stroke risk overall (RR 0.75, 95% CI 0.66–0.86) without increasing mortality, providing stronger support for their role in cerebrovascular prevention (25). Small-interfering RNA-based therapy has further expanded therapeutic options. Inclisiran reduces hepatic PCSK9 synthesis and achieves durable LDL-C lowering with infrequent dosing, which may improve long-term adherence in clinical practice (26). However, this point needs to be stated carefully: while LDL-C lowering within clisiran is well established, its benefit on hard cardiovascular outcomes has not yet been confirmed in a completed large- scale randomized outcomes trial. Ongoing studies such as ORION-4 are expected to clarify whether this durable lipid-lowering effect translates into fewer major cardiovascular events, including stroke. For patients with high cumulative LDL-C burden, statin intolerance, or difficulty sustaining control over time, such long-acting strategies may be especially relevant because cumulative exposure depends not only on biological efficacy but also on treatment persistence.

#### 5.1.3 Lp(a) as a residual atherothrombotic risk factor and emerging RNA-targeted therapies

Beyond LDL-C, lipoprotein(a) [Lp(a)] should also be incorporated into stroke risk assessment and precision lipid management frameworks. Current evidence suggests that elevated Lp(a) is associated with an increased risk of ischemic stroke, with this association appearing more pronounced in younger patients and in large-artery atherosclerotic stroke (27, 28). Emerging evidence also suggests a possible role for remnant cholesterol in residual cerebrovascular risk beyond LDL-C, although current evidence remains less robust than that for Lp(a). Among patients with prior stroke or transient ischemic attack (TIA), higher Lp(a) levels have also been linked to stroke recurrence and worse functional outcomes (29). Because Lp(a) is largely genetically determined and remains relatively stable throughout life, at least one lifetime measurement is recommended in clinical practice to identify additional residual risk.

RNA-targeted therapies for Lp(a), including pelacarsen, olpasiran, zerlasiran, and lepodisiran, have shown marked and sustained Lp(a)-lowering effects in early-phase studies, suggesting potential clinical value (30–33). However, current evidence remains limited to surrogate endpoint improvement, and no completed randomized outcomes trial has yet demonstrated a reduction in stroke incidence or recurrence. Ongoing phase 3 trials, such as Lp(a)HORIZON and OCEAN(a)-Outcomes, include stroke-related endpoints and are expected to clarify whether substantial Lp(a) lowering translates into clinical benefit. Until such results are available, Lp(a) should be regarded as an important stroke risk-enhancing factor and a promising therapeutic target, rather than an intervention target already validated by stroke outcomes trials (34).

### 5.2 Balancing ischemic benefit and hemorrhagic risk

As LDL-C lowering becomes more intensive, a central clinical question emerges: how far should LDL-C be lowered in patients who may also carry hemorrhagic vulnerability? The answer is unlikely to be uniform across all stroke populations.

#### 5.2.1 Identifying hemorrhagic-risk phenotypes

Current evidence does not support a simple conclusion that intensive LDL-C lowering uniformly increases intracerebral hemorrhage (ICH) risk. Instead, concern appears greatest in selected phenotypes, particularly patients with prior lobar ICH, probable cerebral amyloid angiopathy (CAA), multiple strictly lobar cerebral microbleeds, or cortical superficial siderosis. APOE epsilon2 and epsilon4 are established susceptibility alleles for CAA-related lobar ICH and may help identify patients with greater hemorrhagic vulnerability (35, 36). Likewise, MRI markers such as microbleed burden and distribution provide clinically meaningful clues about underlying small-vessel pathology and future ICH risk (37, 38).

However, available quantitative data suggest that caution is warranted once LDL-C is reduced to very low levels in selected patients. A 2021 meta-analysis reported a significantly increased risk of hemorrhagic stroke when LDL-C levels fell below 55 mg/dL, and a 2025 Mendelian randomization study further suggested that lower LDL-C itself, rather than statin exposure per se, may contribute to intracerebral hemorrhage susceptibility (39, 40). These findings do not justify abandoning intensive LDL-C lowering in all patients, but they do support a more individualized risk–benefit assessment in those with prior lobar ICH, probable cerebral amyloid angiopathy, or a substantial burden of strictly lobar cerebral microbleeds (39).

#### 5.2.2 Dynamic risk stratification

A fixed LDL-C target is unlikely to capture the complexity of balancing recurrent ischemic risk against hemorrhagic vulnerability. A more realistic approach would incorporate cumulative LDL-C burden, stroke mechanism, plaque phenotype, prior hemorrhage history, MRI markers of small-vessel disease, blood pressure control, age, and concurrent antithrombotic therapy. Such a framework could move beyond a uniform “lower is always better” paradigm and instead estimate the net clinical benefit of different treatment intensities in different phenotypes.

Future multimodal prediction tools, potentially based on machine learning or Bayesian modeling, may improve this process. Still, these tools will require transparent validation, robust handling of missing data, and clinically interpretable outputs before they can guide routine treatment decisions.

#### 5.2.3 Implementing stratified treatment pathways

In practice, patients with high ischemic risk and low hemorrhagic concern may reasonably be treated according to current intensive LDL-C-lowering paradigms, including combination therapy where needed. By contrast, inpatients with prior lobar ICH, probable CAA, or marked lobar microbleed burden,a more stepwise strategy may be appropriate. Such an approach might begin with careful control of blood pressure and other vascular risks, followed by moderate- intensity statin therapy or ezetimibe-based treatment (41, 42), with periodic clinical and imaging reassessment. If ischemic risk remains substantial, escalation to combination therapy can be considered through individualized shared decision-making rather than by automatic adherence to a single numerical target.

### 5.3 Toward clinically implementable precision lipid management

Precision lipid management in stroke should now move from conceptual framing toward clinically implementable pathways. In current practice, the most pragmatic approach may be to combine repeated LDL-C measurements or a time-weighted exposure metric with stroke mechanism, prior vascular events, hemorrhagic-risk markers, treatment tolerance, and expected adherence, thereby identifying patients most likely to benefit from earlier intensification or longer-acting therapy.

Implementation will also depend on care pathways that support serial lipid measurement, structured follow-up, adherence assessment, and periodic treatment reassessment rather than one-time target setting alone. In this context, agents such as bempedoic acid (30) and evinacumab (43), and other emerging options may expand treatment choices for selected patients, but their place in stroke care will need to be defined within subtype-specific and risk-stratified algorithms.

## 6 Future perspectives

Beyond near-term clinical implementation, the following section highlights research-oriented directions that may refine, validate, or operationalize the cumulative LDL-C burden framework overtime.

### 6.1 Mendelian genetics and causal inference

Mendelian randomization has strengthened the concept that lifelong LDL-C lowering reduces atherosclerotic risk, but stroke-specific inference remains more nuanced. For example, PCSK9- related variants show clear associations with coronary risk reduction, whereas associations with ischemic stroke are weaker and less consistent, likely reflecting limited statistical power, etiologic heterogeneity of ischemic stroke, and differences between lifelong genetic exposure and later pharmacologic intervention (44, 45). These nuances should not be overinterpreted as contradictions; rather, they underscore the complexity of translating genetic exposure models into stroke medicine.

### 6.2 Imaging correlates of cumulative LDL-C exposure

Imaging can serve as a phenotypic bridge between cumulative LDL-C burden and clinical stroke risk. High-resolution carotid and intracranial vessel-wall MRI can identify lipid-rich necrotic core, intraplaque hemorrhage, fibrous cap status, remodeling, and enhancement - features that may reflect prolonged atherogenic injury (46, 47). PET-based imaging may add information about inflammatory activity, whereas radiomic analysis may improve discrimination of high-risk intracranial plaques in specialized research settings (48). Integrating such imaging markers with longitudinal LDL-C exposure may eventually allow a more individualized assessment of vascular vulnerability.

### 6.3 Predictive modeling and artificial intelligence

Artificial intelligence is increasingly used in stroke prediction and outcome modeling, but translation into practice remains limited by data quality, missingness, bias, and explainability (49, 50). In the context of cumulative LDL-C exposure, the most promising future models are likely to be multimodal - combining longitudinal lipid trajectories, clinical covariates, stroke subtype, and imaging markers. Such models should be seen as decision-support tools rather than replacements for clinical judgment.

## 7 Conclusion

Cumulative LDL-C exposure offers a more biologically and clinically informative framework for cerebrovascular risk assessment than single time-point lipid measurement. Available evidence supports its association with ischemic stroke, particularly atherosclerotic stroke, and with recurrent vascular events after stroke. This relationship is mechanistically plausible through chronic effects on plaque vulnerability, endothelial and blood–brain barrier integrity, inflammation, and thrombosis. Clinically, the concept reinforces the importance of early and sustained LDL-C lowering while also highlighting the need for individualized treatment intensity in patients with competing hemorrhagic vulnerability. Future progress will depend on better measurement of cumulative exposure and on integrating lipid burden with genetics, imaging, and dynamic risk prediction.

## References

[1] Feigin VL Brainin M Norrving B Martins SO Pandian J Lindsay P . World stroke organization: global stroke fact sheet 2025. Int J Stroke. (2025) 20:132–44. doi: 10.1177/17474930241308142, 39635884 PMC11786524

[2] Tang J Zhou G Shi S Lu Y Cheng L Xiang J . Systematic analysis of the burden of ischemic stroke attributable to high LDL-C from 1990 to 2021. Front Neurol. (2025) 16:1547714. doi: 10.3389/fneur.2025.1547714, 40255889 PMC12005992

[3] Borén J Chapman MJ Krauss RM Packard CJ Bentzon JF Binder CJ . Low-density lipoproteins cause atherosclerotic cardiovascular disease: pathophysiological, genetic, and therapeutic insights: a consensus statement from the european atherosclerosis society consensus panel. Eur Heart J. (2020) 41:2313–30. doi: 10.1093/eurheartj/ehz962, 32052833 PMC7308544

[4] Golubnitschaja O Polivka J Potuznik P Pesta M Stetkarova I Mazurakova A . The paradigm change from reactive medical services to 3PM in ischemic stroke: a holistic approach utilising tear fluid multi-omics, mitochondria as a vital biosensor and AI-based multi-professional data interpretation. EPMA J. (2024) 15:1–23. doi: 10.1007/s13167-024-00356-6, 38463624 PMC10923756

[5] Ference BA Braunwald E Catapano AL. The LDL cumulative exposure hypothesis: evidence and practical applications. Nat Rev Cardiol. (2024) 21:701–16. doi: 10.1038/s41569-024-01039-5, 38969749

[6] Zhang Y Pletcher MJ Vittinghoff E Clemons AM Jacobs DR Allen NB . Association between cumulative low-density lipoprotein cholesterol exposure during young adulthood and middle age and risk of cardiovascular events. JAMA Cardiol. (2021) 6:1406–13. doi: 10.1001/jamacardio.2021.3508, 34550307 PMC8459309

[7] Ference BA Ginsberg HN Graham I Ray KK Packard CJ Bruckert E . Low-density lipoproteins cause atherosclerotic cardiovascular disease. 1. Evidence from genetic, epidemiologic, and clinical studies. A consensus statement from the european atherosclerosis society consensus panel. Eur Heart J. (2017) 38:2459–72. doi: 10.1093/eurheartj/ehx144, 28444290 PMC5837225

[8] Dai L Xu J Zhang Y Wang A Chen Z Mo J . Cumulative burden of lipid profiles predict future incidence of ischaemic stroke and residual risk. Stroke Vasc Neurol. (2021) 6:581–8. doi: 10.1136/svn-2020-000726, 33827914 PMC8717800

[9] Kang K Wang Y Wu J Wang A Zhang J Xu J . Association between cumulative exposure to increased low-density lipoprotein cholesterol and the prevalence of asymptomatic intracranial atherosclerotic stenosis. Front Neurol. (2020) 11:555274. doi: 10.3389/fneur.2020.555274, 33324314 PMC7726214

[10] Kitagawa K Hosomi N Nagai Y Kagimura T Ohtsuki T Maruyama H . Cumulative effects of LDL cholesterol and CRP levels on recurrent stroke and TIA. J Atheroscler Thromb. (2019) 26:432–41. doi: 10.5551/jat.45989, 30318492 PMC6514170

[11] Lau K-K Chua BJ Ng A Leung IY-H Wong Y-K Chan AH-Y . Low-density lipoprotein cholesterol and risk of recurrent vascular events in Chinese patients with ischemic stroke with and without significant atherosclerosis. J Am Heart Assoc. (2021) 10:e021855. doi: 10.1161/JAHA.121.021855, 34369170 PMC8475056

[12] Lee M Cheng C-Y Wu Y-L Lee J-D Hsu C-Y Ovbiagele B. Association between intensity of low-density lipoprotein cholesterol reduction with statin-based therapies and secondary stroke prevention: a meta-analysis of randomized clinical trials. JAMA Neurol. (2022) 79:349–58. doi: 10.1001/jamaneurol.2021.5578, 35188949 PMC8861901

[13] Leng X Hurford R Feng X Chan KL Wolters FJ Li L . Intracranial arterial stenosis in caucasian versus Chinese patients with TIA and minor stroke: two contemporaneous cohorts and a systematic review. J Neurol Neurosurg Psychiatry. (2021) 92:590–7. doi: 10.1136/jnnp-2020-325630, 33785575 PMC8142447

[14] Jiang H Zhou Y Nabavi SM Sahebkar A Little PJ Xu S . Mechanisms of oxidized LDL-mediated endothelial dysfunction and its consequences for the development of atherosclerosis. Front Cardiovasc Med. (2022) 9:925923. doi: 10.3389/fcvm.2022.925923, 35722128 PMC9199460

[15] Xu S Ilyas I Little PJ Li H Kamato D Zheng X . Endothelial dysfunction in atherosclerotic cardiovascular diseases and beyond: from mechanism to pharmacotherapies. Pharmacol Rev. (2021) 73:924–67. doi: 10.1124/pharmrev.120.000096, 34088867

[16] Lakhan SE Kirchgessner A Tepper D Leonard A. Matrix metalloproteinases and blood-brain barrier disruption in acute ischemic stroke. Front Neurol. (2013) 4:32. doi: 10.3389/fneur.2013.00032, 23565108 PMC3615191

[17] Lin Y-L Chang H-C Chen T-L Chang J-H Chiu W-T Lin J-W . Resveratrol protects against oxidized LDL-induced breakage of the blood-brain barrier by lessening disruption of tight junctions and apoptotic insults to mouse cerebrovascular endothelial cells. J Nutr. (2010) 140:2187–92. doi: 10.3945/jn.110.123505, 20980646

[18] Wu Y-C Bogale TA Koistinaho J Pizzi M Rolova T Bellucci A. The contribution of β-amyloid, tau and α-synuclein to blood–brain barrier damage in neurodegenerative disorders. Acta Neuropathol. (2024) 147:39. doi: 10.1007/s00401-024-02696-z, 38347288 PMC10861401

[19] Berger M Naseem KM. Oxidised low-density lipoprotein-induced platelet hyperactivity—receptors and signalling mechanisms. Int J Mol Sci. (2022) 23:9199. doi: 10.3390/ijms23169199, 36012465 PMC9409144

[20] Hu Y Li Y Luo Y Wang N Zheng Y. Lectin-like oxidized low-density lipoprotein receptor-1 (LOX-1): a potential therapeutic target in ischemic stroke. Transl Stroke Res. (2024) 16:1412–23. doi: 10.1007/s12975-024-01307-z, 39576412 PMC12202518

[21] Amarenco P Kim JS Labreuche J Charles H Abtan J Béjot Y . A comparison of two LDL cholesterol targets after ischemic stroke. N Engl J Med. (2020) 382:9–19. doi: 10.1056/NEJMoa1910355, 31738483

[22] Giugliano RP Pedersen TR Park J-G Ferrari GMD Gaciong ZA Ceska R . Clinical efficacy and safety of achieving very low LDL- cholesterol concentrations with the PCSK9 inhibitor evolocumab: a prespecified secondary analysis of the FOURIER trial. Lancet. (2017) 390:1962–71. doi: 10.1016/S0140-6736(17)32290-0, 28859947

[23] Sabatine MS Giugliano RP Keech AC Honarpour N Wiviott SD Murphy SA . Evolocumab and clinical outcomes in patients with cardiovascular disease. N Engl J Med. (2017) 376:1713–22. doi: 10.1056/NEJMoa1615664 28304224

[24] Gaba P O’Donoghue ML Park J-G Wiviott SD Atar D Kuder JF . Association between achieved low-density lipoprotein cholesterol levels and long-term cardiovascular and safety outcomes: an analysis of FOURIER-OLE. Circulation. (2023) 147:1192–203. doi: 10.1161/CIRCULATIONAHA.122.063399, 36779348

[25] Moustafa B Oparowski D Testai S Guman I Trifan G. Efficacy and safety of PCSK9 inhibitors for stroke prevention: systematic review and meta- analysis. J Stroke Cerebrovasc Dis. (2024) 33:107633. doi: 10.1016/j.jstrokecerebrovasdis.2024.107633, 38336118

[26] Zhang Y Chen H Hong L Wang H Li B Zhang M . Inclisiran: a new generation of lipid-lowering siRNA therapeutic. Front Pharmacol. (2023) 14:1260921. doi: 10.3389/fphar.2023.1260921, 37900173 PMC10611522

[27] Arnold M Schweizer J Nakas CT Schütz V Westphal LP Inauen C . Lipoprotein(a) is associated with large artery atherosclerosis stroke aetiology and stroke recurrence among patients below the age of 60 years: results from the BIOSIGNAL study. Eur Heart J. (2021) 42:2186–96. doi: 10.1093/eurheartj/ehab081, 33709115

[28] Koh MY Toh KZ Loh ED Teo YN Joon KC Tan QX . Association of elevated lipoprotein(a) levels with ischemic stroke in young patients - a systematic review and meta-analysis. J Stroke Cerebrovasc Dis. (2024) 33:107960. doi: 10.1016/j.jstrokecerebrovasdis.2024.107960, 39222699

[29] Palaiodimou L Melanis K Stefanou M-I Theodorou A Giannopoulos S Lambadiari V . The association of lipoprotein(a) and stroke recurrence: a systematic review and meta-analysis. J Stroke. (2025) 27:161–8. doi: 10.5853/jos.2024.04623, 40494575 PMC12152458

[30] Nissen SE Linnebjerg H Shen X Wolski K Ma X Lim S . Lepodisiran, an extended-duration short interfering RNA targeting lipoprotein(a): a randomized dose-ascending clinical trial. JAMA. (2023) 330:2075–83. doi: 10.1001/jama.2023.21835, 37952254 PMC10641766

[31] Nissen SE Wang Q Nicholls SJ Navar AM Ray KK Schwartz GG . Zerlasiran—a small-interfering RNA targeting lipoprotein(a): a phase 2 randomized clinical trial. JAMA. (2024) 332:1992–2002. doi: 10.1001/jama.2024.21957, 39556769 PMC11574722

[32] O’Donoghue ML Rosenson RS Gencer B López JAG Lepor NE Baum SJ . Small interfering RNA to reduce lipoprotein(a) in cardiovascular disease. N Engl J Med. (2022) 387:1855–64. doi: 10.1056/NEJMoa2211023, 36342163

[33] Tsimikas S Karwatowska-Prokopczuk E Gouni-Berthold I Tardif J-C Baum SJ Steinhagen-Thiessen E . Lipoprotein(a) reduction in persons with cardiovascular disease. N Engl J Med. (2020) 382:244–55. doi: 10.1056/NEJMoa1905239, 31893580

[34] Cho L Nicholls SJ Nordestgaard BG Landmesser U Tsimikas S Blaha MJ . Design and rationale of lp(a)HORIZON trial: assessing the effect of lipoprotein(a) lowering with pelacarsenon major cardiovascular events in patients with CVD and elevated lp(a). Am Heart J. (2025) 287:1–9. doi: 10.1016/j.ahj.2025.03.019, 40185318

[35] Biffi A Anderson CD Jagiella JM Schmidt H Kissela B Hansen BM . APOE genotype and extent of bleeding and outcome in lobar intracerebral haemorrhage: a genetic association study. Lancet Neurol. (2011) 10:702–9. doi: 10.1016/S1474-4422(11)70148-X, 21741316 PMC3153411

[36] Greenberg SM Charidimou A. Diagnosis of cerebral amyloid angiopathy. Stroke. (2018) 49:491–7. doi: 10.1161/STROKEAHA.117.016990, 29335334 PMC5892842

[37] Greenberg SM Ziai WC Cordonnier C Dowlatshahi D Francis B Goldstein JN . 2022 guideline for the management of patients with spontaneous intracerebral hemorrhage: a guideline from the American Heart Association/American Stroke Association. Stroke. (2022) 53:E282–E361. doi: 10.1161/STR.0000000000000407 35579034

[38] Kozberg MG Yi I Freeze WM Auger CA Scherlek AA Greenberg SM . Blood–brain barrier leakage and perivascular inflammation in cerebral amyloid angiopathy. Brain Commun. (2022) 4:fcac245. doi: 10.1093/braincomms/fcac245, 36267331 PMC9576155

[39] Masson W Lobo M Siniawski D Masson G Lavalle-Cobo A Molinero G. LDL-C levels below 55 mg/dl and risk of hemorrhagic stroke: a meta- analysis. J Stroke Cerebrovasc Dis. (2021) 30:105655. doi: 10.1016/j.jstrokecerebrovasdis.2021.105655, 33571878

[40] Shi S Zheng Z Chen W Song Y Dou K. Low density lipoprotein cholesterol but not statins is the direct cause of intracerebral hemorrhage. Eur J Pharmacol. (2025) 998:177443. doi: 10.1016/j.ejphar.2025.177443 40023359

[41] Bohula EA Wiviott SD Giugliano RP Blazing MA Park J-G Murphy SA . Prevention of stroke with the addition of ezetimibe to statin therapy in patients with acute coronary syndrome in IMPROVE-IT (improved reduction of outcomes: Vytorin efficacy international trial). Circulation. (2017) 136:2440–50. doi: 10.1161/CIRCULATIONAHA.117.029095, 28972004

[42] Cannon CP Blazing MA Giugliano RP McCagg A White JA Theroux P . Ezetimibe added to statin therapy after acute coronary syndromes. N Engl J Med. (2015) 372:2387–97. doi: 10.1056/NEJMoa1410489, 26039521

[43] Raal FJ Rosenson RS Reeskamp LF Hovingh GK Kastelein JJP Rubba P . Evinacumab for homozygous familial hypercholesterolemia. N Engl J Med. (2020) 383:711–20. doi: 10.1056/NEJMoa2004215, 32813947

[44] Davies NM Holmes MV Smith GD. Reading mendelian randomisation studies: a guide, glossary, and checklist for clinicians. BMJ. (2018) 362:K601. doi: 10.1136/bmj.k601, 30002074 PMC6041728

[45] Hopewell JC Malik R Valdés-Márquez E Worrall BB Collins R Metastroke Collaboration Of The Isgc. Differential effects of PCSK9 variants on risk of coronary disease and ischaemic stroke. Eur Heart J. (2018) 39:354–9. doi: 10.1093/eurheartj/ehx373, 29020353 PMC5837489

[46] Brinjikji W Huston J Rabinstein AA Kim G-M Lerman A Lanzino G. Contemporary carotid imaging: from degree of stenosis to plaque vulnerability. J Neurosurg. (2016) 124:27–42. doi: 10.3171/2015.1.JNS142452, 26230478

[47] Mandell DM Mossa-Basha M Qiao Y Hess CP Hui F Matouk C . Intracranial vessel wall MRI: principles and expert consensus recommendations of the American society of neuroradiology. AJNR Am J Neuroradiol. (2017) 38:218–29. doi: 10.3174/ajnr.A4893, 27469212 PMC7963837

[48] Shi Z Zhu C Degnan AJ Tian X Li J Chen L . Identification of high-risk plaque features in intracranial atherosclerosis: initial experience using a radiomic approach. Eur Radiol. (2018) 28:3912. doi: 10.1007/s00330-018-5395-1, 29633002 PMC6081255

[49] Amann J Blasimme A Vayena E Frey D Madai VI. Explainability for artificial intelligence in healthcare: a multidisciplinary perspective. BMC Med Inform Decis Mak. (2020) 20:310. doi: 10.1186/s12911-020-01332-6, 33256715 PMC7706019

[50] Bonkhoff AK Grefkes C. Precision medicine in stroke: towards personalized outcome predictions using artificial intelligence. Brain. (2022) 145:457–75. doi: 10.1093/brain/awab439, 34918041 PMC9014757

